# Predictors for mechanical ventilation and short-term prognosis in patients with Guillain-Barré syndrome

**DOI:** 10.1186/s13054-015-1123-2

**Published:** 2015-11-17

**Authors:** Jingyan Yang, Linpei Jia

**Affiliations:** Department of Anesthesiology, Cangzhou Central Hospital, Cangzhou, Hebei Province China; Department of Nephrology, the Second Hospital of Jilin University, Changchun, Jilin Province China

Wu and colleagues retrospectively analyzed the clinical data of 541 patients who were diagnosed with Guillain-Barré syndrome (GBS) from 2003 to 2014, among whom 80 patients (14.8 %) required mechanical ventilation [1]. Via multivariate logistic regression analysis, independent predictors for mechanical ventilation and short-term prognosis in mechanically ventilated patients were identified [1]. This study was well designed and conducted. In-hospital infections were not mentioned in the study, however, which seems lacking for such a study given it is an important parameter.

We wonder whether the rate of in-hospital infections was simply not included in the analysis or whether it was proved not to be an independent predictor for mechanical ventilation after multivariate logistic regression analysis. Ali and colleagues [2] described a historical cohort of mechanically ventilated patients with GBS in a tertiary-care center. They found that ventilator-associated pneumonia was the most frequent complication and was associated with prolonged mechanical ventilation [2]. We are eager, therefore, to know about the incidence of ventilator-associated pneumonia and whether it is a predictor for poor short-term outcome. In addition, intravenous corticosteroids are not recommended for the treatment of GBS according to several international guidelines and we would like to know why corticosteroids were used on a large number of patients with GBS in the study by Wu and colleagues.

GBS typically occurs after an infectious disease, two-thirds of patients presenting with symptoms of a respiratory or gastrointestinal tract infection before its onset [3]. Wu and colleagues included antecedent infections as a parameter in their analysis; however, we are eager to know whether stratified analysis according to respiratory and gastrointestinal infections was performed.

## Abbreviations

GBS: Guillain-Barré syndrome
IVIg: intravenous immunoglobulin

## References

[1] Wu X, Li C, Zhang B, Shen D, Li T, Liu K (2015). Predictors for mechanical ventilation and short-term prognosis in patients with Guillain-Barré syndrome. Crit Care..

[2] Ali MI, Fernández-Pérez ER, Pendem S, Brown DR, Wijdicks EF, Gajic O (2006). Mechanical ventilation in patients with Guillain-Barré syndrome. Respir Care..

[3] van den Berg B, Walgaard C, Drenthen J, Fokke C, Jacobs BC, van Doorn PA (2014). Guillain-Barré syndrome: pathogenesis, diagnosis, treatment and prognosis. Nat Rev Neurol..

[4] Saravu K, Preethi V, Kumar R, Guddattu V, Shastry AB, Mukhopadhyay C (2013). Determinants of ventilator associated pneumonia and its impact on prognosis: a tertiary care experience. Indian J Crit Care Med..

[5] Hughes RA, van Doorn PA (2012). Corticosteroids for Guillain-Barré syndrome. Cochrane Database Syst Rev..

[6] The Dutch Guillain-Barré Study Group (1994). Treatment of Guillain-Barré syndrome with high-dose globulins combined with methylprednisolone: a pilot study. Ann Neurol.

[7] van Koningsveld R, Schmitz PI, Meché FG, Visser LH, Meulstee J, van Doorn PA (2004). Effect of methyprednisolone when added to standard treatment with intravenous immunoglobulin for Guillain-Barré syndrome: randomized trial. Lancet.

[8] Hughes RA (2004). Treatment of Guillain-Barré syndrome with corticosteroids: lack of benefit?. Lancet..

