# Supplementary material for: Predictors for mechanical ventilation and short-term prognosis in patients with Guillain-Barré syndrome
Source: Crit Care. 2015 Nov 17;19:407. doi: 10.1186/s13054-015-1123-2 (PMC4650284; doi:10.1186/s13054-015-1123-2)
Supplement: Related texts

# Reply: Authors’ response

**Authors:** Xiujuan Wu, Kangding Liu, Hong-Liang Zhang

We appreciate Yang and Jia’s comments on our study. As they mentioned, GBS is usually triggered by antecedent infections. In the retrospective study, however, stratified analysis was not performed to address whether different antecedent infections could serve as a predictor for mechanical ventilation and short-term prognosis. The overall incidence of in-hospital infections for the patients with GBS was approximately 19.6 % in our study. The incidence was 85.8 % in mechanically ventilated patients, most of which were ascribed to ventilator-associated pneumonia, and was significantly lower in those without respiratory assistance (11.4 %, *P* < 0.01). Thus, we did not include in-hospital infections in predicting mechanical ventilation due to their inherent causal relationship. This was also the case when we searched for predictors for short-term prognosis of patients who required mechanical ventilation. However, the poor short-term prognosis of mechanically ventilated patients might be related to ventilator-associated pneumonia, which may potentially increase the duration of mechanical ventilation [4].

As to the therapeutic values of corticosteroids in treating GBS, they were proved ineffective when administered alone [5]. Nevertheless, a study found that 76 % of patients who received intravenous immunoglobulin (IVIg) combined with corticosteroids improved by at least one grade on the GBS disability scale compared with 53 % patients in the IVIg-treated group [6]. The median time to regain unaided walking was also shorter in the combination therapy group [6]. Similar results were found when adjustments were made in a randomized double-blind, placebo-controlled and multicentre study [7]. Thus, intravenous corticosteroids as an add-on therapy have been used in the treatment of severe or protracted GBS cases [7]. However, we found that add-on use of intravenous corticosteroids was a risk factor for poor short-term prognosis in mechanically ventilated patients who were severely paralyzed. This might either be due to its harmful effects on denervated muscles or its inhibition of macrophage functions in the reparative and regenerative processes of the peripheral nerves [8] or be related to the increased incidence of in-hospital infections and hyperglycemia caused by intravenous use of corticosteroids [4, 5].
